# Evaluation of the Activity of the Essential Oil from an Ornamental Flower against *Aedes aegypti*: Electrophysiology, Molecular Dynamics and Behavioral Assays

**DOI:** 10.1371/journal.pone.0150008

**Published:** 2016-02-29

**Authors:** Patrícia C. Bezerra-Silva, Kamilla A. Dutra, Geanne K. N. Santos, Rayane C. S. Silva, Jorge Iulek, Paulo Milet-Pinheiro, Daniela M. A. F. Navarro

**Affiliations:** 1 Laboratório de Ecologia Química, Departamento de Química Fundamental, Universidade Federal de Pernambuco, 50670–901, Recife, PE, Brazil; 2 Laboratório de Purificação e Determinação de Estruturas de Proteínas, Departamento de Química, Universidade Estadual de Ponta Grossa, 84070–900, Ponta Grossa, PR, Brazil; Centro de Pesquisas René Rachou, BRAZIL

## Abstract

Dengue fever has spread worldwide and affects millions of people every year in tropical and subtropical regions of Africa, Asia, Europe and America. Since there is no effective vaccine against the dengue virus, prevention of disease transmission depends entirely on regulating the vector (*Aedes aegypti*) or interrupting human-vector contact. The aim of this study was to assess the oviposition deterrent activity of essential oils of three cultivars of torch ginger (*Etlingera elatior*, Zingiberaceae) against the dengue mosquito. Analysis of the oils by gas chromatography (GC)—mass spectrometry revealed the presence of 43 constituents, of which α-pinene, dodecanal and *n*-dodecanol were the major components in all cultivars. Solutions containing 100 ppm of the oils exhibited oviposition deterrent activities against gravid *Ae*. *aegypti* females. GC analysis with electroantennographic detection indicated that the oil constituents *n*-decanol, 2-undecanone, undecanal, dodecanal, *trans*-caryophyllene, (E)-β-farnesene, α-humulene, *n*-dodecanol, isodaucene and dodecanoic acid were able to trigger antennal depolarization in *Ae*. *aegypti* females. Bioassays confirmed that solutions containing 50 ppm of *n*-dodecanol or dodecanal exhibited oviposition deterrent activities, while a solution containing the alcohol and aldehyde in admixture at concentrations representative of the oil presented an activity similar to that of the 100 ppm oil solution. Docking and molecular dynamics simulations verified that the interaction energies of the long-chain oil components and *Ae*. *aegypti* odorant binding protein 1 were quite favorable, indicating that the protein is a possible oviposition deterrent receptor in the antenna of *Ae*. *aegypti*.

## Introduction

Dengue is a vector-borne disease that occasionally develops into the potentially fatal complication known as dengue hemorrhagic fever [1]. This viral disease affects millions of people every year on all continents, but most especially in tropical and subtropical areas [2]. According to the Brazilian Ministry of Health [3] there were 745,957 cases of dengue fever in Brazil between 1st January and 18th April 2015, an incidence 234% higher than that reported for the same period in 2014, and 229 hemorrhagic fever-related deaths were recorded. Based on these data, dengue has attained epidemic status in Brazil according to the classification of the World Health Organization [4].

The dengue virus is spread by the bite of an infective female *Aedes aegypti* mosquito, a mode of transmission that is similar to that of Yellow fever and Chikungunya viruses [5]. Since there is no effective vaccine against the dengue virus, prevention of disease transmission depends entirely on regulating the vector or interrupting human-vector contact [2]. Various methods are available for controlling *Ae*. *aegypti*, for example, the use of compounds that prevent females from feeding, reproducing or ovipositing, or the application of agents that kill, or inhibit the development of eggs or larvae [6,7].

The most effective compounds employed commonly in governmental programs aimed at controlling mosquitoes are either synthetic insecticides, such as organophosphates (e.g. temephos), or natural toxins (e.g. *Bacillus thuringiensis israelensis* toxin). However, a number of studies have shown that continuous application of synthetic organic insecticides can give rise to the development of resistant populations of *Ae*. *aegypti* [8–10]. Furthermore, the use of such pesticides has been called into question because of their adverse effects on human health and the environment [11–13]. These concerns have alerted the scientific community to the urgent need of seeking alternative technologies for vector control.

One approach to reduce the number of mosquitoes and the level of viral transmission in an urban environment involves the application of attractants for mass trapping or repellents [14]. This strategy works because mosquitoes use cues in order to locate mates, nectar sources, hosts for blood feeding and sites for oviposition [15]. Moreover, adult *Ae*. *aegypti* females require at least one blood meal for completion of the gonadotropic cycle, hence disease transmission necessitates the fulfillment of one oviposition cycle before viral transfer can occur in a subsequent blood meal [16].

Current knowledge suggests that the localization of sites involves a behavioral response by the female mosquito to one or more stimuli including visual cues, moisture, heat, carbon dioxide and other chemical emanations. However, it appears that the most important mechanism by which mosquitoes locate their hosts centers on the detection of chemical signals [15]. In human skin, for example, compounds such as L-lactic acid have been identified as chemical attractants of *Ae*. *aegypti* females [17]. In addition, it has been shown that attractiveness is enhanced by carbon dioxide and ammonia [18], both of which are found in human breath and skin.

From a physiological perspective, the reception of chemical signals by insects is generally restricted to olfactory structures, mainly the antennae and maxillary palps [19,20], and is assisted by odorant-binding proteins (OBPs) [21]. These low-molecular weight proteins comprise nanostructures that encapsulate hydrophobic ligands and carry the water-soluble OBP–semiochemical complexes through the sensillar lymph surrounding the odorant receptors [19]. Currently, 114 genes coding for OBPs were already identified in *Ae*. *aegypti* [22]; four antennae-specific OBPs were isolated of which OBP1 is the major one and the only with the 3D structure available [20,21]. A more complete understanding of these systems may help in the search for active substances, and a number of approaches, including molecular field analysis, molecular docking and evaluation of system dynamics, have been employed in order to address this issue [23,24].

Numerous plant-derived substances have been screened in order to find natural compounds that could be applied in the control of *Ae*. *aegypti*. Such studies have focused mainly on the evaluation of larvicidal and insecticidal properties, but repellent, ovicidal, pupicidal and oviposition deterrent activities have also been assessed [6,25–28]. The aims of the present work were: (i) to evaluate the potential deterrent effect of the essential oil of the ornamental flower torch ginger (*Etlingera elatior*) against *Ae*. *aegypti*; (ii) to identify, through gas chromatographic (GC) analysis with electroantennographic detection (EAD), the specific oil constituents responsible for EAD activity; and (iii) to confirm the presence of receptors for these constituents on the antennae of female mosquitoes.

## Materials and Methods

### Chemicals

All chemicals and solvents used in the study were of Analytical Grade or of higher purity. Dodecanal and *n*-dodecanol were purchased from Sigma-Aldrich (Gillingham, UK) and were used as received.

### Maintenance of *Ae*. *aegypti*

A colony of *Ae*. *aegypti* was maintained in the laboratory at 28 ± 1°C under a relative humidity of 70 ± 5% and a photoperiod of 14L:10D. Adult mosquitoes were maintained on a 10% sucrose solution, while females were also blood fed on pigeons (1h) four days after emergence. The sucrose solution was taken off the cage a day prior to the blood feeding. This assay was previously authorized by the Ethical Committee of the Federal University of Pernambuco.

### Plant material

Fresh inflorescences of red torch, pink torch and porcelain cultivars of *E*. *elatior* (Jack) R.M. Smith (Zingiberaceae) were obtained from a commercial grower (Florix Flora Tropical, Recife, PE, Brazil) in April 2012. The plant material was authenticated by Jefferson Rodrigues Maciel (Jardim Botânico do Recife, Recife, PE, Brazil), and a voucher specimen was deposited in the herbarium of the Jardim Botânico do Recife with identification number J.R. MACIEL 1654.

### Preparation of essential oil

Floral bracts (ca. 500 g of each cultivar) were comminuted in a blender and hydrodistilled for 3 h in a Clevenger-type apparatus. The essential oil layer was separated, dried over anhydrous sodium sulfate and stored in a hermetically sealed amber-glass vial at -5°C until required for assay. The yield of oil was reported as the quotient of the mass of oil collected and the fresh weight of plant material extracted [29].

### Analysis of essential oil by gas chromatography-mass spectrometry (GC-MS)

Essential oil constituents were identified by GC-MS analysis performed on an Agilent Technologies (Palo Alto, CA, USA) 5975C Series GC/MSD quadrupole instrument equipped with an Agilent J&W non-polar DB-5 fused silica capillary column (30 m × 0.25 mm i.d.; film thickness 0.25 μm). The analytical conditions were: sample (1 μL) injected in split mode (50:1) with injector temperature at 250°C; oven temperature held initially at 40°C for 2 min, then increased to 230°C at 4°C/min and held at 230°C for 5 min; helium carrier gas flow maintained at 1 mL/min at a constant pressure of 7.0 psi; mass selective detector source and quadrupole temperatures set to 230°C and 150°C, respectively; MS obtained at 70 eV and recorded in the range 35–350 *m/z* at 1.0 scan/s.

Individual components of the essential oil were tentatively identified by comparison of retention indices, obtained by co-injection of sample with C_9_–C_30_ linear hydrocarbons and calculated according to the Van den Dool and Kratz equation [30],[29] with those reported in the literature. The MS data acquired for each component were matched with those stored in the mass spectral library of the GC–MS system (MassFinder 4, NIST08 and Wiley Registry™ 9th Edition) and with published spectra [31] in order to confirm identity.

### Electrophysiological analysis

Electrophysiological analyses were performed with the aim of detecting constituents of the essential oil of *E*. *elatior* with the potential to be perceived by *Ae*. *aegypti* females. The instrumentation comprised a Thermo Scientific (Milan, Italy) Trace^™^ Ultra GC equipped with a flame ionization detector (FID), a Syntech (Kirchzarten, Germany) EAD with heated transfer line and two-channel universal serial bus acquisition controller, and a VICI Metronics (Poulsbo, WA, USA) VB-5-ValcoBond^®^ capillary column (30 m x 0.25 mm i.d.; 0.25 μm film thickness) [32]. The column outlet was coupled to two lengths of deactivated capillary (40 cm x 0.25 mm i.d.) via an SGE Analytical Science (Trajan, Melbourne, VIC, Australia) splitter tee. One capillary led to the FID while the other was routed outside the GC oven and into a glass tube where the effluent was mixed with a clean and humidified airflow and directed over the head preparation. The flow of helium carrier gas through the column was maintained at 1 mL/min by electronic control and nitrogen make-up gas was added immediately before the splitter. The injector temperature was set at 200°C, and an aliquot (0.5 μl) of a headspace sample of essential oil was injected in splitless mode with the oven temperature of 60°C. After 1 min, the injector split valve was opened and the oven temperature was increased to 200°C at 7°C/min and held at 200°C for 5 min.

Ten female mosquitoes (10 to 20 days old) were used in the analyses three days after a blood meal. The head of each individual was excised from the thorax with a scalpel and the tips of the antennae were cut. The base of the head and the tip of both antennae were then mounted between two glass capillary electrodes filled with insect ringer solution (8.0 g/L sodium chloride, 0.4 g/L potassium chloride, 0.4 g/L calcium chloride), and two silver wire electrodes were inserted into each capillary, thereby closing the electrical circuit with the head preparation. An essential oil constituent was considered EAD-active when it elicited a depolarization response in at least four individual head preparations.

### Oviposition deterrent activity assay

In order to evaluate the oviposition deterrent activity of the essential oils, female *Ae*. *aegypti* mosquitoes were subjected to standard choice assays. Test solutions containing 100 ppm of essential oil or 50 ppm of *n*-dodecanol or dodecanal were prepared by dissolving 0.02 g of oil or 0.01 g of the standard compound in six drops of Tween^®^ 80 and diluting to a final volume of 200 mL with water. Test solutions were diluted further with water where necessary. Negative control solutions were prepared in the same manner but without oil or standard compound. Two disposable cups were placed at diagonally opposite corners of a cage measuring 33 × 21 × 30 cm, with one cup containing 25 mL of test solution and the other 25 mL of negative control solution. For paired assays, one cup contained 100 ppm of oil solution and the other a mixture of the two standard compounds in amounts equivalent to those present in the oil solution, i.e. *n*-dodecanol (25 ppm) and dodecanal (50 ppm) Filter papers were placed on the internal surface of each cup in order to provide support for oviposition. Ten gravid insects were transferred to the cage, which was then maintained at 28 ± 1°C and 70 ± 5% relative humidity for 16 h in the dark. Oviposition response was determined at the end of the assay period by counting the numbers of eggs laid on each of the filter papers. Each assay was replicated eight times and the mean values obtained for each of the test samples were compared using Student’s t-test at an alpha level of 0.05 [33].

### Molecular docking and dynamics of constituents of *E*. *elatior* oil to *Ae*. *aegypti* OBP 1

Molecular formulae and initial structural coordinates of the long chain components present in the essential oils of *E*. *elatior* were obtained with the aid of BIOVIA Draw software (Biovia, San Diego, CA, USA; http://accelrys.com/products/informatics/cheminformatics/draw/) and VEGA ZZ molecular modeling toolkit (http://nova.disfarm.unimi.it/cms/index.php?Software_projects:VEGA_ZZ) [34]. Tripos mol2 format (http://www.tripos.com/data/support/mol2.pdf) files with coordinates were submitted to the antechamber module [35] of the AMBER package [36] in order to estimate AM1-BCC charges [37] and assign general AMBER force field (GAFF) atom types [38], each of which were subsequently inspected individually. Structures were energy minimized in a periodical TIP3 water box with edges at least 12 Å from any non-water atoms using the sander module of the AMBER package [36]. Docking was performed using the Autodock Vina program (http://vina.scripps.edu/index.html) [39] in which the *Ae*. *aegypti* odorant binding protein 1 [20] (AaegOBP1, PDB ID: 3K1E) was the receptor, immersed in a box with dimensions 120 × 120 × 120 Å. Sites for molecular interaction were searched throughout the whole protein surface in blind docking mode with search exhaustiveness set to 128. Molecular dynamics simulations were applied to the five best poses for each ligand in order to estimate binding energy. In these calculations, the GAFF force field [38] was applied to the ligands, the Amber ff99SB protein force field [40] was applied to the protein receptor and the TIP3 model was used for water molecules [41]. Initially, each docked complex was immersed in a water box with edges at least 8 Å from any non-water atoms and sufficient counter-ions were put in place of solvent molecules in order to neutralize the total charge of the system. Subsequently, the following steps were carried out: i) minimization of system energy, with strong restraints on the receptor + ligand positions, until convergence; ii) minimization of complete system until convergence; iii) heating of system from 0 to 300 K over 50 ps with weak restraints on the receptor + ligand positions; iv) short dynamics for 50 ps with weak restraints on the receptor + ligand positions; and v) exploration dynamics for 500 ps. In all cases, a cut-off of 12 Å was applied to all non-bonded interactions. One thousand snapshots of each trajectory were taken at equal intervals and one hundered of them were subjected to MM/GBSA methodology [42] in order to estimate the binding free energy Δ*G*.

## Results and Discussion

The yields of essential oils obtained by hydrodistillation of inflorescences of red torch, pink torch and porcelain cultivars of *E*. *elatior* were 0.094, 0.052 and 0.049% (w/w), respectively. Similar oil yields have been reported previously from inflorescences of *E*. *elatior* [43] and from inflorescences of another member of the Zingiberaceae, namely *Alpinia purpurata* [44]. Forty-three components were identified in *E*. *elatior* oil by GC–MS analysis (Table 1), of which fifteen were present in the oils of all three cultivars, three were detected only in the red torch cultivar, eleven in the pink torch cultivar and two in the porcelain cultivar.

**Table 1:** Relative proportions of the constituents of essential oils obtained from red torch, pink torch and porcelain cultivars of *E*. *elatior*.

No.	Compound^a^	Retention indices		Content (% of total oil)		
		Determined^b^	Literature^c^	Red torch	Pink torch	Porcelain
**1**	**α-Pinene**	932	939	7.83	22.98	2.55
**2**	**Camphene**	948	954	0.05	0.19	-
**3**	**Thuja-2,4(10)-diene**	954	960	0.04	0.16	-
**4**	**β-Pinene**	976	979	0.55	2.56	0.20
**5**	**β-Myrcene**	991	990	0.29	1.50	-
**6**	**p-Cymene**	1025	1026	0.04	0.14	-
**7**	**Limonene**	1028	1029	0.38	0.96	0.09
**8**	**β-(*Z*)-Ocimene**	1038	1037	0.06	0.25	0.06
**9**	**β-(*E*)-Ocimene**	1049	1050	0.06	0.29	0.07
**10**	**γ-Terpinene**	1058	1059	-	0.03	-
**11**	**α-Terpineol**	1190	1188	-	0.09	0.06
**12**	**Terpinolene**	1088	1088	0.03	0.16	-
**13**	**Pinene oxide**	1099	1099	0.03	0.03	-
**14**	**α-Campholenal**	1126	1126	-	0.05	-
**15**	**Undecane**	1102	1100	0.03	-	-
**16**	**Decanal**	1206	1201	0.98	1.44	2.99
**17**	***n*-Decanol**	1272	1269	0.17	0.52	0.65
**18**	**2-Undecanone**	1294	1294	0.08	0.18	0.35
**19**	**Methyl myrtenate**	1298	1294	-	0.04	-
**20**	**Undecanal**	1307	1306	0.11	0.15	0.12
**21**	***n*-Undecanol**	1373	1370	0.02	0.05	-
**22**	**9-Decenyl acetate**	1399	1399	0.03	-	-
**23**	***trans*-Sobrerol**	1377	1374	-	0.07	-
**24**	**β-Elemene**	1393	1390	-	0.08	-
**25**	**Dodecanal**	1410	1408	49.37	25.70	57.73
**26**	***trans*-Caryophyllene**	1422	1419	0.42	0.56	2.40
**27**	**(E)-β-Farnesene**	1459	1456	-	4.40	0.45
**28**	**α-Humulene**	1460	1454	1.99	-	-
**29**	***n*-Dodecanol**	1476	1470	31.53	24.05	24.58
**30**	**β-Selinene**	1491	1490	-	0.25	0.21
**31**	**α-Zingiberene**	1499	1493	-	1.06	-
**32**	**2-Tridecanone**	1499	1495	-	-	0.23
**33**	**Isodaucene**	1503	1500	0.18	0.38	-
**34**	**α-Farnesene**	1503	1505	0.65	-	0.16
**35**	**β-Bisabolene**	1512	1505	-	0.08	-
**36**	**γ-(*Z*)-Bisabolene**	1520	1515	-	0.07	-
**37**	**δ-Cadinene**	1528	1523	-	0.06	-
**38**	**Dodecanoic acid**	1565	1566	0.09	0.66	1.24
**39**	**β-Caryophyllene epoxide**	1586	1583	-	-	0.20
**40**	**Dodecyl acetate**	1609	1607	3.15	4.86	2.86
**41**	**Citronellyl angelate**	1660	1657	-	0.17	-
**42**	***n*-Tetradecanol**	1676	1672	1.50	3.61	1.53
**43**	***n*-Heptadecane**	1699	1700	-	0.08	-
	**Alcohols**			33.22 (4)^d^	28.23 (4)	26.76 (3)
	**Aldehydes**			50.46 (4)	27.29 (3)	60.84 (3)
	**Esters**			3.18 (2)	4.86 (1)	2.86 (1)
	**Fatty acids**			0.09 (1)	0.66 (1)	1.24 (1)
	**Hydrocarbons**			0.03 (1)	0.08 (1)	-
	**Ketones**			0.08 (1)	0.18 (1)	0.58 (2)
	**Monoterpenes**			9.25 (8)	28.92 (9)	3.03 (6)
	**Oxygenated monoterpenes**			0.11 (3)	0.58 (7)	-
	**Sesquiterpenes**			3.24 (4)	6.94 (9)	3.22 (4)
	**Oxygenated sesquiterpenes**			-	0.17 (1)	0.20 (1)
	**Total**			**99.66**	**97.91**	**98.73**

- Not detected.

^a^ Constituents listed in order of elution from a non-polar DB-5 column

^b^ Retention indices calculated from retention times in relation to those of a series of C_9_-C_30_ *n*-alkanes on a 30 m DB-5 capillary column

^c^ Values taken from Adams^30^

^d^ Values in parenthesis indicate the number of compounds in the class.

The volatiles of the inflorescences of *E*. *elatior* were rich in long chain alcohols and aldehydes and contained a range of mono- and sesquiterpenes. Qualitatively, the oils of the three cultivars were not substantially different, but there were significant variations in the relative proportions of the constituents even though dodecanal, *n*-dodecanol and α-pinene were the major components in all cases. The overall oil composition shown in Table 1 is in accordance with that described by Zoghbi and Andrade [45] for this species, although these authors did not specify the cultivar employed and the specimens were collected in the northern region of Brazil. In contrast, Jaafar et al. [46] reported that 1,1-dodecanediol diacetate, cyclododecane and α-pinene were the major constituents of *E*. *elatior* collected in Penang, Malaysia. This difference could be related to dissimilar edaphic conditions or to variations in the populations studied. Relationships between such conditions and oil yield or composition have been described for the essential oils of various species including *Salvia officinalis* [47], *Cymbopogon winterianus* [48] and *Coriandrum sativum* [49]. We have reported previously that the ornamental flowers of two cultivars of the Zingiberaceous species *A*. *purpurata* also presented differences in their essential oils [44].

**Fig 1:**
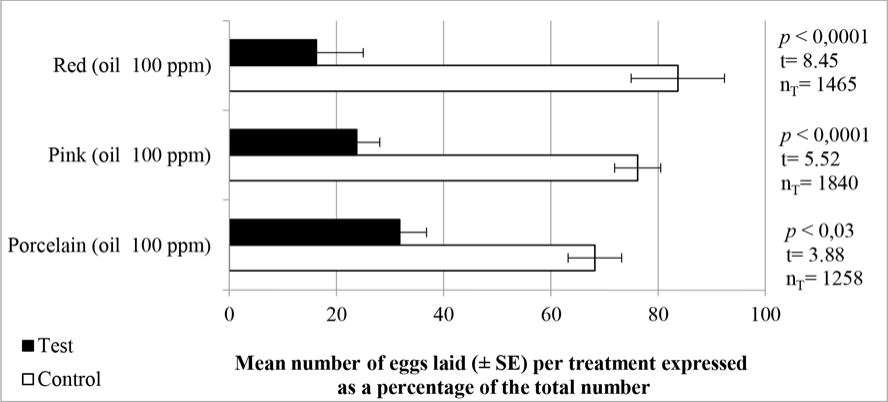
Mean percentages (± SE) of eggs laid by *Ae*. *aegypti* females in choice assays. Cups filled with a solution of an essential oil from a cultivar of *E*. *elatior* (100 ppm) or with control solution. Each assay involved 10 gravid insects and was replicated at least 6 times. n_T_ = total number of eggs laid.

As shown in Fig 1, the essential oils of *E*. *elatior* cultivars showed oviposition deterrent effects at 100 ppm (*P* < 0.05) against *Ae*. *aegypti* in that the numbers of eggs laid in cups containing oil solutions were significantly lower (30% or less) than those oviposited in control cups. The choice of oviposition site by a gravid female mosquito is determined by several factors. Potential sites are initially identified on the basis of visual and olfactory cues [16], following which short-range cues become increasingly important in subsequent selection. Such cues include temperature and chemical signals received by contact chemoreceptors distributed along the body of the mosquito [16]. Electrophysiological studies on *Ae*. *aegypti* have revealed that, while the blood meal is being digested, neurons susceptible to host-produced cues, such as lactic acid, become less sensitive, while neurons susceptible to oviposition site attractants, such as methyl butyrate, become more sensitive [50]. Accordingly, when oviposition deterrents are detected, few, if any, eggs are laid at that site [16]. In the present study, eggs laid in cups containing oil solution were placed preferentially on the outside of the support, thereby signifying the reluctance of females to lay eggs adjacent to the solution or the source of volatiles.

Few reports are available concerning the effectiveness of essential oils, or their individual components, as mosquito oviposition deterrents [27,51–54]. However, the oviposition activities of the oils of *E*. *elatior* against *Ae*. *aegypti* established in the present study are similar to those previously reported for two Zingiberaceous species, namely *A*. *purpurata* [44] and *A*. *galanga* [55]. Regarding the activities of individual constituents of the oil, it is important to note that the process of isolation and purification of specific components can be time consuming and expensive by virtue of the complex nature of the oil and the low concentrations of many of the constituents. For this reason, the development of techniques that would allow active components to be identified without prior isolation is of considerable importance [56].

**Fig 2:**
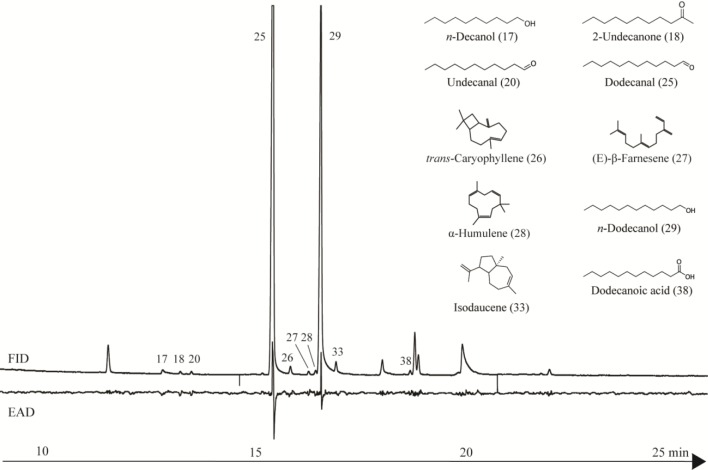
FID and EAD chromatograms of essential oil of *E*. *elatior* recorded concomitantly during GC separation. The EAD-active peaks 17, 18, 20, 25–29, 33 and 38 were identified as *n*-decanol, 2-undecanone, undecanal, *n*-dodecanal, *trans*-caryophyllene, (E)-β-Farnesene, α-humulene, *n*-dodecanol, isodaucene and dodecanoic acid, respectively (see Table 1).

In the present study, we have integrated for the first time the technique of GC-EAD with bioassays in order to identify the individual compounds within a complex oil that are responsible for oviposition deterrent effects against *Ae*. *aegypti*. The essential oil from the red torch cultivar of *E*. *elatior* was selected for GC-EAD analysis since it presented the highest oviposition deterrent activity. Ten components of the oil triggered antennal depolarization in *Ae*. *aegypti* females, and these were identified as *n*-decanol, 2-undecanone, undecanal, dodecanal, *trans*-caryophyllene, (E)-β-Farnesene, α-humulene, *n*-dodecanol, isodaucene and dodecanoic acid (Fig 2). However, dodecanal and *n*-dodecanol elicited the most pronounced antennal depolarization and, since these two compounds accounted for almost 80% of the total oil content, they were submitted to bioassay in order to test their significance as oviposition deterrents.

**Fig 3:**
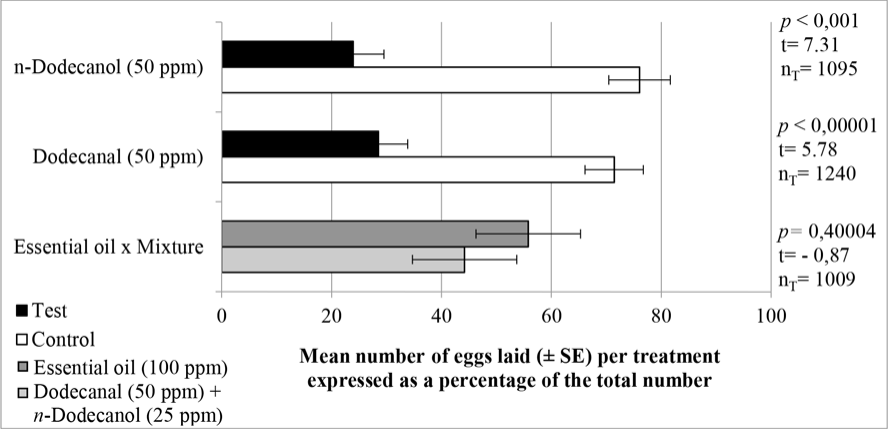
Mean percentages (± SE) of eggs laid by *Ae*. *aegypti* females in choice assays. The cups contained: **upper plot**—a solution of dodecanal (50 ppm) and control solution; **middle plot**—a solution of *n*-dodecanol (50 ppm) and control solution; **lower plot**—a solution containing essential oil of red torch cultivar (100 ppm) and a solution containing dodecanal (50 ppm) and *n*-dodecanol (25 ppm) in admixture. Each assay involved 10 gravid insects and was replicated at least 6 times. n_T_ = total number of eggs laid.

Choice assays performed with solutions containing commercial standards of dodecanal and *n*-dodecanol clearly showed that both compounds possessed oviposition deterrent activity (Fig 3). Thus, in comparison with the controls, gravid females laid fewer eggs in cups containing 50 ppm dodecanal (28.5% of total laid) or 50 ppm *n*-dodecanol (23.9% of total laid). However, in order to confirm the role of these components in the overall deterrent activity of the essential oil, paired tests were performed in which one cup was filled with a solution containing 100 ppm of oil while the second held a mixed solution containing dodecanal (50 ppm) and *n*-dodecanol (25 ppm), such concentrations representing their respective proportions in the oil of red torch cultivar. No significant differences were observed between the numbers of eggs laid in the cups containing the oil and the standard compounds in admixture (Fig 3). These findings provide strong evidence that the two components of the essential oil are mainly responsible for its oviposition deterrent activity.

Previous studies involving the GC-EAD technique, but without associated bioassays, have demonstrated that a range of compounds are capable of eliciting responses in the antennae of mosquitoes. Thus, Campbell et al. [56] reported that a number of mono- and sesquiterpenes, including *trans*-caryophyllene, α-terpineol, β-pinene, germacrene-D, limonene and α-zingiberene, present in essential oils were able to stimulate the antennae of *Ae*. *aegypti*. Additionally, the sesquiterpenes α-curcumene, β-sesquiphellandrene, zingiberene and β-bisabolene from the essential oil of ginger (*Zingiber officinale*, Zingiberaceae) [57], *trans*-caryophyllene from *Ocimum forskolei* [58] and limonene from *Panicum maximum* [59] reportedly stimulate the antennae of *Ae*. *aegypti* females. Long chain compounds present in the oil of *E*. *elatior*, particularly the aldehydes undecanal and dodecanal that have also been found in human odor exhalation [15,60], have been reported to stimulate the antenna of other mosquito species including *Culex quinquefasciatus* [61]. Although their capacities to repel or deter oviposition have not been determined, earlier studies have shown that unsaturated fatty acids [62] and aliphatic carboxylic acids [63] exhibit deterrent activity against *C*. *quinquefasciatus*, whereas tetradecanoic acid is repellent to both *Ae*. *aegypti* and *C*. *quinquefasciatus* [64].

Although only dodecanal and *n*-dodecanol were assayed for oviposition deterrent activity in the present study, other long chain oxygenated compounds, such as dodecanoic acid, undecanal, 2-undecanone and *n*-decanol, elicited responses in the antennae of *Ae*. *aegypti* females. Such responses may well be due to OBPs present in the sensillar of the antennae, a possibility supported by the computational models studied in this work.

**Fig 4:**
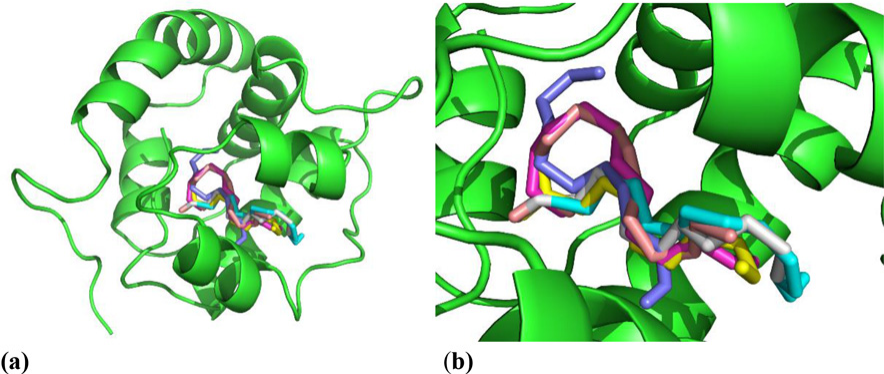
The best docked poses for the long chain ligands to *Ae*. *aegypti* OBP 1. **(a)** view of the whole protein structure; **(b)** view of the binding site magnified from **(a)**.

A number of three dimensional structures of OBPs bound to long chain compounds have already been determined including PDB ID: 3K1E [20], 3OGN [65], 3R1O [66] and 3V2L [67]. The blind docking calculations relating to the binding of dodecanal, *n*-dodecanol, dodecanoic acid, undecanal, 2-undecanone and *n*-decanol to *Ae*. *aegypti* OBP 1 performed in the present study showed the clear preference of these ligands for the long tunnel present in the protein structure. Fig 4A and 4B show only the first best (for clarity) docked pose for these ligands, but all five best-scored poses for each of the ligands occupied this tunnel. Molecular dynamics simulations indicated the permanence of the ligands in this site, and mean Δ*G* values (calculated according to MM/GBSA methodology) of -2463, -2430, -2431, -2437, -2459 and -2469 kcal/mol for the ligands in the order listed above were quite favorable for binding. These results suggest that OBP 1 may be one of the receptors in the antennae of *Ae*. *aegypti* that binds these volatiles when the female is at the stage of oviposition site selection.

## Conclusions

The essential oils of three cultivars of *E*. *elatior* exhibited oviposition deterrent activity against *Ae*. *aegypti* females. Although ten components of the complex oil elicited responses in female antennae, the major constituents, namely dodecanal and *n*-dodecanol, produced the most pronounced responses. Commercial standards of the long chain aldehyde and alcohol were themselves active in oviposition deterrent bioassays, and a mixture containing these compounds in proportions equivalent to those found in the essential oil was found to be as active as the oil itself. Docking and molecular dynamics calculations showed that these compounds bind to OBP 1 and may play an important role in oviposition site-seeking behavior, thus indicating that the protein is a possible oviposition deterrent receptor in the antenna of *Ae*. *aegypti*.

## Abbreviations

EAD: electroantennographic detection
FID: flame ionization detection
GAFF: general AMBER force field
GC: gas chromatography
MS: mass spectrometry
OBP: odorant binding protein
